# Comparison of pre-hospital emergency services time intervals in patients with heart attack in Arak, Iran

**DOI:** 10.5249/jivr.v13i1.1614

**Published:** 2021-01

**Authors:** Abed Khanizade, Davoud Khorasani-Zavareh, Soheila Khodakarim, Mohammad Palesh

**Affiliations:** ^*a*^ Department of Health Services Management, Virtual School of Medical Education and Management, Shahid Beheshti University of Medical Sciences, Tehran, Iran.; ^*b*^ Workplace Health Promotion Research Center (WHPRC), Shahid Beheshti University of Medical Sciences, Tehran, Iran.; ^*c*^ Department of Health in Emergencies and Disasters, School of Public Health and Safety, Shahid Beheshti University of Medical Sciences, Tehran, Iran.; ^*d*^ School of Medicine, Shiraz University of Medical Sciences, Shiraz, Iran.; ^*e*^ Department of Health Services Management, Virtual School of Medical Education and Management, Shahid Beheshti University of Medical Sciences, Tehran, Iran.

**Keywords:** Prehospital, Time intervals, Emergency Medical, Services, Heart attack

## Abstract

**Background:**

After cardiac arrest, the possibility of death or irreversible complications will highly increase in the absence of cardiac resuscitation within 4 to 6 minutes. Accordingly, measuring the pre-hospital services time intervals is important for better management of emergency medical services delivery. The purpose of this study then was to investigate pre-hospital time intervals for patients with heart attack in Arak city, based on locations and time variables.

**Methods:**

This is a retrospective descriptive cross-sectional study, which was conducted at the Arak Emergency Medical Services (EMS) during 2017-2018. Data were analyzed by SPSS version 13.

**Results:**

The total number of heart attack patients registered in Arak emergency medical services was 2,659 of which 51% of patients were males. Six percent of patients were under 25 and about 49 percent were between 46 and 65 years old. The average of activation, response, on-scene, transportation, recovery and total time intervals were 3:30, 7:56, 15:15, 13:34, 11:07, 12:11, and 41:25, respectively. In the city area, the shortest and longest average response time intervals were in spring and winter, respectively. In out of the city area, the shortest average response time interval was in summer and the longest one in autumn. The shortest and the longest average response time intervals in the city area were in June and March, respectively, and in out of the city area, the shortest average response time interval was in June and the longest one in April.

**Conclusions:**

The shorter response and delivery time interval compared to the other studies may indicate improvement in the provision of EMS. Special attention should be paid to the facilities and equipment of vehicles during cold seasons to be in the shortest possible time. Also, training and informing the staff more about the code of cardiac patients along with general public education can help improve these intervals.

## Introduction

Cardiovascular disease is the first cause of death, worldwide. According to the World Health Organization (WHO), about 17.7 million people die as a result of cardiovascular disease each year; which represents 31% of all deaths in the world.^1^ According to the WHO, Iran with more than 9,000 cases of cardiovascular disease per 100,000 people was one of the countries with the highest cardiovascular disease rate in the world. Among all cardiovascular diseases, ischemic heart disease with about 26% of total deaths and 11% of the total burden of disease in 2015 was the first cause of death and disability adjusted life years (DALY) in Iran.^2^ Also, according to the Ministry of Health and Medical Education of Iran, about 17.3% of deaths in Markazi province occur due to heart disease.^3^

There is growing clinical evidence showing that air pollution is associated with increasing in the cardiovascular disease mortality, hospital admission, and cardiac arrest.^4,5^ Also a study conducted in Greece showed a relationship between air pollution and ischemic heart disease.^6^ Arak is the capital city of the Markazi province located in the central Iran. Due to intense industrial activities, urbanization, and an increase in the number of motor vehicles in the last decades, air pollution has had an ascending trend in Arak city. A study conducted in Arak reported that ambient air pollution is associated with hospital admission due to cardiovascular disease in Arak city.^7^

Emergency Medical Services (EMS) is the arrangement of personnel, facilities, and equipment for the effective and coordinated delivery of EMS required in the prevention and management of incidents, which occur either as a result of a medical emergency, incidents as well as natural disasters.^8^

The time-sensitive nature of the emergency medical services (EMS) can be shown with cardiac emergency care systems, meaning that the chance of survival of cardiac arrest without care outside of the hospital, will reduce by 10% per minute. Moreover, there is good evidence that demonstrates the importance of providing early defibrillation (in less than 4 minutes) for patient survival.^9^ The goals set by the American Heart Association, include an increase in the percentage of patients with heart attack reaching to a hospital in the first hour from the onset of symptoms, to 20%, and in six hours after symptoms, to 90%.^10^ In acute conditions of cardiovascular disease, especially the ischemic condition, reducing the time of receiving primary care is important for improving the prognosis of patients; however, there is little guidance on how the emergency medical system can optimize their time before reaching the hospital.^11^ The current guidelines of the American College of Cardiology and the American Heart Association suggest that the time between the first medical call and the time of the definitive treatment of a heart attack should be less than 90 minutes.^12^

In emergency medical services, there are various time intervals, including response time, on-scene time and transport time. Many researchers emphasize the reduction of pre-hospital times of medical emergencies to use golden hours that are important in the survival of patients.^13,14^ Studying the factors affecting the transport delay of patients in the emergency medical services can improve the efficiency of the system, for example, by improving response time and transport time in remote areas or areas with limited facilities that increase the transportation time.^15^ So far, limited studies have been conducted in the pre-hospital time intervals of patient with cardiac diseases in Iran, which have only described them. Due to the high volume of air pollution in Arak city and its impact on heart diseases, and also Arak is an industrial city with different climate, from hot and dry in summer and cold and humid in winter, examining pre-hospital time intervals at different times like seasons and months can help to identify shortcoming in the EMS service delivery. Based on the mentioned points, this study was aimed to determine the status of intervals of pre-hospital emergency services for patients with heart attack in Arak city, based on location and various time intervals.

## Materials and Methods

This study is a retrospective descriptive cross-sectional study that examines the pre-hospital time intervals of heart attack patients in Arak city from 21 March 2017 to 20 March 2018.

**Study Area and Study Population**

This study was carried out in Arak city, which is the capital of Markazi province, in the center of Iran, with an area of 7,178 km2, consisting of three parts, i.e., Central, Sarough, and Masumiyeh. According to the census of 2016, the population of the city of Arak city, was 591,756. The model of the pre-hospital emergency system used in Arak as well as throughout Iran is the Anglo-American type, which the main goal of this model provides essential medical services and rapid transfer of patients to the hospital. The emergency medical posts in Arak city include eight city and nine road posts, which are covered by 98 personnel and 17 ambulances.

**Data Collection Method**

In this study, data recorded in the statistics unit of the Arak emergency medical services center, where the data are recorded accurately and correctly with routine quality assessment, were obtained based on the standard questionnaire developed by the Ministry of Health and Medical Education of Iran. This questionnaire includes demographic information, including name, age, sex, main patient complaints, and the time of service delivery.

The time intervals in this study include activation interval, response interval, on-scene interval, transportation interval, delivery interval, recovery interval, and total pre-hospital interval.

**Data Source and Sample Selection**

Trained medical personnel, record medical information of heart attack patients on special forms, and all of this information is collected in the central computer of statistics unit in Arak EMS. In this study, by census sampling method, all missions registered at the dispatch center of Arak EMS during 2017-2018, with a diagnosis of heart attack disease which ambulance dispatched to serve the patient, were included in the study. The total number of missions registered at the dispatch center in the Arak emergency medical services during the study period was 29,723 cases, of which 2,812 were patients with heart attacks. All patients who had been diagnosed with a medical emergency, but not as a heart attack were excluded. Moreover, information about patients transmitted by the air emergency, which was less than one percent, has not been included in this study Missions in which, patients with a heart attack diagnosis refused to be transferred to a hospital, were calculated only for activation, response, on-scene and return time intervals. According to the inclusion and exclusion criteria, a census was performed and in total 2,659 patients were included in the study.

**Data Analysis**

Descriptive statistics of different pre-hospital intervals, including activation interval, response interval, on-scene interval, transportation interval, delivery interval, recovery interval, and total pre-hospital interval on the mean, median, mode, maximum and minimum, were analyzed. Accordingly, the Kruskal-Wallis test was used to compare the average of intervals in different seasons and months. Missing data regarding all intervals were less than 10%. Version 13 SPSS was used to analyze the data. In all tests, the significance level for P-Value was less than 0.05.

**Ethical Considerations**

First of all, permission was obtained by the Arak University of Medical Sciences to conduct the study, then explained the research objectives to the Arak Emergency Medical Services administrator. Also, the agreement to conduct the research was obtained from the ethics committee of Shahid Beheshti University of Medical Sciences (ethical code: IR.SMBU.PHNS.REC.1397.22). In addition to ethical considerations, names and other personal information were removed from the data.

## Results

The total number of registered missions in Arak Emergency Medical Services (EMS) was 29,723, of which 2,659 were diagnosed with heart attack. These patients are further categorized by age, sex, location, season, and month.

The number of patients with heart attack registered in Arak EMS was 2,659. The number of missions carried out in out of the city location was about 3 times higher than the number of out of the city missions.

In terms of gender distribution, the number of male patients (51%) was slightly higher than female patients (48%). Also, according to the age group, divided into five categories, the highest number of patients was in the age range of 46-65 years (38.9%) and the least number of patients was in the age range of >25 years (6.1%).

The shortest mean time interval was the activation time interval (3:30) (minutes and seconds). The mean of response time, on-scene time, transportation time, delivery time, and recovery time in minutes and seconds were 7:56, 15:15, 13:34, 11:07, and 12:11, respectively, and finally, the mean of total time interval as the longest time interval was 41:25 (minutes and seconds).

All pre-hospital time intervals for missions out of the city are longer than missions in the city, except for the on-scene time interval in the fall season. In missions in the city, the shortest intervals were in the spring season.

There was a significant difference between the mean recovery, transportation, and total time intervals in missions in the city in different seasons of the year, and in missions out of the city, there was only a significant difference between the average recovery interval in different seasons.

In missions in the city, the shortest response time, on-scene time, and transportation time intervals were in June. Comparison of time intervals in different months revealed that between the mean of transportation, recovery, and total time intervals within missions in the city, and, between the on-scene and total time intervals in missions out of the city there was a significant difference in different months of the year.

## Discussion

The findings of this study showed that the ratio of male and female patients was close and the majority of the patients were men. In a study in Japan on the relationship between pre-hospital time intervals of heart patients and their outcomes, as well as a study carried out in California, 54.1% and 65% of patients were men, respectively. The mean age of heart attack patients in these studies was 76 and 62 years.^12,16^ The first indicator of pre-hospital emergency services is the activation time interval, which is the time from receipt of the call by the emergency service personnel until the ambulance departs from its post.

**Prehospital Time Intervals**

**Activation Interval**

The average activation time interval in studies conducted in Saudi Arabia and North America, examining the pre-hospital time intervals were less than our finding, that is, 1:35 (minutes and seconds) and 1 minute, respectively.^17,18^ Compared to those studies, the activation time interval for patients with heart attack in Arak was longer. It appears that a shorter activation time interval in the emergency medical services will cause a better response time interval. A long activation time can have personnel, managerial, cultural, structural, and environmental reasons. Perhaps, as the activation time interval is longer, pre-hospital emergency deployment personnel will face many challenges, such as stress, high-speed driving, and other hazards to reduce the response time interval.^19^ It is also important to note that detecting the location of the incident is influential in providing a faster and more appropriate response. Currently, the location of the incident is not identified during calls the emergency service, while, according to the World Health Organization's emphasis and other studies, the importance of this issue has been emphasized.^20^

**Response Interval**

The average response interval, which is considered to be the most important pre-hospital time interval in terms of EMS performance, in this study was shorter than the mean obtained in the study conducted in Shiraz, Iran that was 10 minutes.^21^ Compared to the standard response time, i.e., below 8 minutes in Iran, the response time interval of patients with heart attack in this study, was acceptable. Many factors may affect the response time, such as the type of ambulances, geographic differences, socio-demographic patterns, public awareness, traffic conditions, weather, and location of the incident.^22-25^ Based on the results of a qualitative study that was conducted among Urmia personnel to identifying factors affecting pre-hospital time intervals, the most important obstacles related to pre-hospital intervals were the involvement of untrained people and urban infrastructure that delayed arriving at the crash scene or during hospital transportation. It is also emphasized that heavy work shifts, shortages in the emergency system, and a low number of personnel at dispatch centers can delay the response to calls.^13^ There has long been a debate over whether the response time is effective on the outcome of patients, and various studies have published controversial results. For example, studies in Japan and Canada found no relationship between response time and mortality rates.^12,26^ On the other hand, many studies, including studies in the United States of America on how to develop a pre-hospital emergency system, the role of response time in pre-hospital emergency cases, and evaluating the performance of the system, emphasized the relationship between response time and patients' outcomes in such a way that the reduction of the response interval can improve the patient's outcomes.^27-29^ However, the instructions in the pre-hospital emergency system are currently emphasizing the response time reduction, which should be less than 8 minutes in 80% of cases. 

**On-Scene Interval**

In study conducted in Arak city, the on-scene time interval was 16 minutes, which is very close to this interval in our study.^30^ Moreover, according to a study in South Korea, which evaluated the relationship between on-scene time interval and the neurological outcomes of patients with myocardial infarction, patients with an on-scene time interval of more than 30 minutes had a lower chance of recovery compared to other patients with a shorter on-scene time interval.^31^ On-scene interval includes access to the patient, initial assessment, medical treatment, relocation, and patient preparation for transfer from the scene.^32^ The number of personnel, personnel skills, and patient conditions are influential factors in this regard. In some conditions, stabilization of patients is beneficial in the scene, and in some critical situations, the patient transfer is more important than stay.^19^ The existence of a systemic view of the phenomenon desired to improve management and reduce time intervals can be helpful, as this systemic perspective has been emphasized in other fields of study.^33^ In studies conducted in Saudi Arabia and Japan, the average on-scene time interval were around 14 and 17 minutes, respectively.^17,34^ Also, according to a study carried out in Denmark in order to examine on-scene time interval and patient outcomes, there was a positive correlation and the mortality rate and on-scene time intervals, of which while on-scene interval was longer than 20 minutes.^35^ In general, the other factors influencing on-scene time interval include the way people interact with the scene of the incident, which has been emphasized in other studies,^36^ indicating the importance of providing general education programs to educate people about the interaction in emergencies.^13^

**Transport Interval**

In a study in Japan, the transport time interval was 27 minutes, and it was found that the increasing mortality rate in heart patients was related to a long transport time interval.^12^ In a study conducted in Arak city, the transport time interval was 11 minutes, which is shorter than this interval in our study.^37^ However, considering national standards, this interval is acceptable.

**Delivery Interval**

The average delivery time interval in this study was desirable by the 15 minutes standard and could be attributed to the proper coordination of the emergency medical services and destination hospital, which in turn is due to the protocol for heart attack patients (247) that would prevent waste of time effectively. The patient's delivery time interval includes the time before triage, the triage time, and the time after the triage. According to a study on the patient's delivery time in the United States, 69 percent of the patient's delivery comes from the time after triage, when staff spends a lot of time for writing the report and waiting for an empty bed. The average overall in-hospital delivery time of the patient in a study by Segal was around 45 minutes, and there was no significant correlation between the patient delivery time and gender, age, and day and night hours.^38^ Also, in a study in Saudi Arabia, the average delivery time interval was 14 minutes.^17^

**Recovery Interval**

The average time of ambulance returning from the hospital and arriving back at the ambulance depot in this study was slightly longer than the recovery time interval in the Saudi Arabia study, lasting 11 minutes, which seems to be appropriate given the wider geographic area that is covered.^17^ Ambulance personnel should return to their location in the shortest time and be ready to perform subsequent missions. Any delay in this part will delay the subsequent missions because in the absence of an ambulance, another ambulance from the nearest location should be deployed for the mission and this will affect the response time of that mission. 

**Total interval**

The total pre-hospital time interval is defined as the time between the initial call receipt by personnel and the arrival of the ambulance to the hospital. This time was 32 minutes for heart attack patients in the California study,^16^ that is shorter than this time interval in our study. Many studies emphasize that the reduction of pre-hospital time intervals, especially those with heart attack, can increase the chance of survival and reduce the complications of the disease. Also, several studies showed that the use of diagnostic tools in the prehospital phase can improve the accuracy of diagnosis and reduce pre-hospital time intervals.^30^ Accordingly, pre-hospital emergency systems have specific time limits for each time interval.

**Pre-Hospital Time Intervals in Seasons and Months**

Comparison of average time intervals of heart attack patients in different seasons revealed that there was a significant difference in missions in the city, between mean transport, recovery, and total pre-hospital intervals, while in missions that are out of the city, there is a significant difference only in the recovery time interval. The shortest time intervals occurred in the spring season and the longest ones in the summer and autumn. In a study that evaluated the effect of the summer on the pre-hospital delay in heart patients and was conducted in the United States, the longest transport and total time intervals were observed throughout the summer. Because the deterioration of the symptoms of cardiac patients is associated with a reduction in treatment time, exposure to higher temperatures during the summer may likely reduce the warning signs and may cause vague heart symptoms and difficulty in diagnosis.^39^ This can contribute to increasing the pre-hospital time intervals, because one of the factors involved in the decisions made by emergency personnel is the patient's condition and symptoms.

Comparison of average time intervals of heart attack patients in different months of the year showed that there was a significant difference in missions in the city between mean transport, recovery, and total time intervals, and in missions out of the city, there was a significant difference between the average of on-scene and total time intervals in different months of the year. The length of the on-scene time interval in March, which is one of the winter months, can be because personnel need more time to prepare the patient for cold-weather transportation.^40^

**Study Strengths and Imitation**

One strengths of the study are its cost effectiveness and quality control of the findings. Focusing on the study limitation, patients were transferred to two hospitals located in different parts of Arak city, and some EMS posts were close to hospitals, while some were from hospitals; therefore, our results may not be generalizable to other EMS and health systems. Because of the retrospective nature of our study, we trusted the recorded data in EMS center, so it is possible some prehospital time intervals may have been underestimated.

## Conclusion

According to the findings of this study, due to the weather conditions in the region, special attention should be paid to the facilities and equipment of vehicles during the cold seasons to fulfill the missions in the shortest possible time. Also, training and informing staff as to the code of cardiac patients along with general public education can help in shortening these time intervals. It is suggested that the efficacy of the protocol developed for reducing the time intervals of cardiac patients should be evaluated by conducting interventional studies.

**Acknowledgments**

The authors thank the Shahid Beheshti University of Medical Sciences, Arak University of Medical Sciences, and the Arak Emergency Medical Services center.
